# Complete and Prolonged Inhibition of Herpes Simplex Virus Type 1 Infection In Vitro by CRISPR/Cas9 and CRISPR/CasX Systems

**DOI:** 10.3390/ijms232314847

**Published:** 2022-11-27

**Authors:** Dmitry S. Karpov, Natalia A. Demidova, Kirill A. Kulagin, Anastasija I. Shuvalova, Maxim A. Kovalev, Ruslan A. Simonov, Vadim L. Karpov, Anastasiya V. Snezhkina, Anna V. Kudryavtseva, Regina R. Klimova, Alla A. Kushch

**Affiliations:** 1 Center for Precision Genome Editing and Genetic Technologies for Biomedicine, Engelhardt Institute of Molecular Biology, Russian Academy of Sciences, Vavilov Str., 32, 119991 Moscow, Russia; 2 N.F. Gamaleya National Research Centre for Epidemiology and Microbiology, Ministry of Health of the Russian Federation, Gamaleya Str., 18, 123098 Moscow, Russia; 3 Engelhardt Institute of Molecular Biology, Russian Academy of Sciences, Vavilov Str., 32, 119991 Moscow, Russia

**Keywords:** herpes simplex virus type 1, HSV-1 *UL30*, CRISPR/Cas9, CRISPR/CasX, Vero cell line

## Abstract

Almost all people become infected with herpes viruses, including herpes simplex virus type 1 (HSV-1), during their lifetime. Typically, these viruses persist in a latent form that is resistant to all available antiviral medications. Under certain conditions, such as immunosuppression, the latent forms reactivate and cause disease. Moreover, strains of herpesviruses that are drug-resistant have rapidly emerged. Therefore, it is important to develop alternative methods capable of eradicating herpesvirus infections. One promising direction is the development of CRISPR/Cas systems for the therapy of herpesvirus infections. We aimed to design a CRISPR/Cas system for relatively effective long-term and safe control of HSV-1 infection. Here, we show that plasmids encoding the CRISPR/Cas9 system from *Streptococcus pyogenes* with a single sgRNA targeting the *UL30* gene can completely suppress HSV-1 infection of the Vero cell line within 6 days and provide substantial protection within 9 days. For the first time, we show that CRISPR/CasX from *Deltaproteobacteria* with a single guide RNA against *UL30* almost completely suppresses HSV-1 infection of the Vero cell line for 3 days and provides substantial protection for 6 days. We also found that the Cas9 protein without sgRNAs attenuates HSV-1 infection. Our results show that the developed CRISPR/Cas systems are promising therapeutic approaches to control HSV-1 infections.

## 1. Introduction

Herpes simplex virus type 1 (HSV-1) belongs to the family *Herpesviridae*. Nearly 100% of the world’s adults are infected with at least one herpesvirus during their lifetime [1]. According to the World Health Organization, about 67% of the world’s population, are infected with HSV-1 [2]. After primary infection, HSV-1 becomes a lifelong latent form. Reactivation of latent HSV-1 can results in recurrent ulcerated blisters on mucosal surfaces near the primary focus of infection (orolabial or genital herpes). Reactivation leading to ocular lesions, such as herpetic epithelial or stromal keratitis, can lead to blindness [3]. In immunocompromised individuals, herpesvirus infections often lead to severe complications, up to death. There is evidence that HSV-1 may be involved in the development of such neuropathologies as Alzheimer’s disease [4], multiple sclerosis [5], meningitis and encephalitis [6]. As other sexually transmitted infections, HSV-1 genital herpes may contribute to the development of infertility, negatively affect pregnancy, up to fetal death, cause congenital and neonatal infections [7,8]. Reactivation of herpesviruses has also been observed in patients infected with SARS-CoV-2 [9,10], the causative agent of the ongoing COVID19 pandemic [11]. The combination of widespread herpesviruses latently infecting humans and the COVID19 pandemic should draw more attention to other viruses that could affect human health. Reactivation of latent herpesviruses can provoke complications after organ transplantation [12,13] or xenotransplantation [14], including a recent heart xenotransplantation from a genetically modified pig [15].

All approved drugs (Acyclovir, Valacyclovir, Ganciclovir, etc.) can only control actively replicating herpesviruses, but cannot eliminate the latent forms [16,17]. A promising class of helicase-primase inhibitors effectively suppresses latent forms of alpha-herpesviruses [18,19,20]. However, side effects prevent their widespread use in the clinic [21,22]. Moreover, immunosuppressed patients can develop drug-resistant variants of HSV-1 and HSV-2 [17]. Strains resistant to helicase-primase inhibitors are also known [23]. These data demonstrate the urgent need to search for alternative effective approaches to control herpesvirus infections.

Genome editing technologies, mainly CRISPR/Cas systems directed against major genes, can control herpesvirus infections [24,25,26]. Delivery of CRISPR/Cas components in retroviral vectors is very efficient, but raises safety concerns due to integration mutagenesis of retroviral vectors [27,28]. Moreover, the most effective antiviral CRISPR/Cas9 systems used two or three spacers [26,29,30]. Since Cas9 has a relatively high level of off-target activity [31], the use of two or more spacers should reduce the safety of the system due to an increase in the number of potential off-targets.

We and others have shown that the CRISPR/Cas9 system can effectively suppress HSV-1 infection being expressed from plasmids [32,33]. The goal of this study is to further improve the efficacy, duration of protection and safety of CRISPR/Cas systems against HSV-1. We show that HSV-1 infection can be completely suppressed by plasmid-expressed CRISPR/Cas9 using only one spacer. Moreover, for the first time we have shown that the smallest and most precise CRISPR/CasX system can also be used to control HSV-1 infection. Our data demonstrate the promise of developing a small, efficient and safer approach to control herpesvirus infections using genome editing technologies.

## 2. Results

### 2.1. Characterization of the Genome HSV-1 Strain

The HSV-1 “US” strain was obtained from the State virus collection of the Gamaleya National Research Center for Epidemiology and Microbiology (Moscow, Russia). The genome of the strain was sequenced using Illumina technology and de novo assembled to produce draft genome. A total of 398 contigs were obtained, N50 = 2345. It is possible that the low N50 is due to DNA fragmentation after thermal inactivation of the virus. Nevertheless, the quality of the draft genome was sufficient for further experiments. Using BLAST, we determined the presence of all 79 genes characteristic of human HSV-1. Next, we searched for the genome of the most similar HSV-1 strains. For this purpose, all publicly available HSV-1 genome assemblies were downloaded from NCBI Virus (https://www.ncbi.nlm.nih.gov/labs/virus/vssi/#/ accessed on 25 September 2022) using TaxID 10298 and prespecifying a genome length between 130,000 and 160,000. The downloaded HSV-1 genomes were compared with the genome of our “US” strain to calculate the average nucleotide identity (ANI) using the USEARCH algorithm [34]. According to the results, the three most similar strains were L2 (ANI = 99.7667, Supplemental Table S1), HSV-1-San-Francisco-USA-1982-H193-CSF (ANI = 99.6848) and v48_d100_oral (ANI = 99.6772). Since L2 is another Russian strain [35], it suggests that the US and L2 strains share some sequence features making them distinct from other strains.

### 2.2. UL30 Identified as an Effective Anti-HSV-1 Target of the CRISPR/Cas9 System

**Figure 1:**
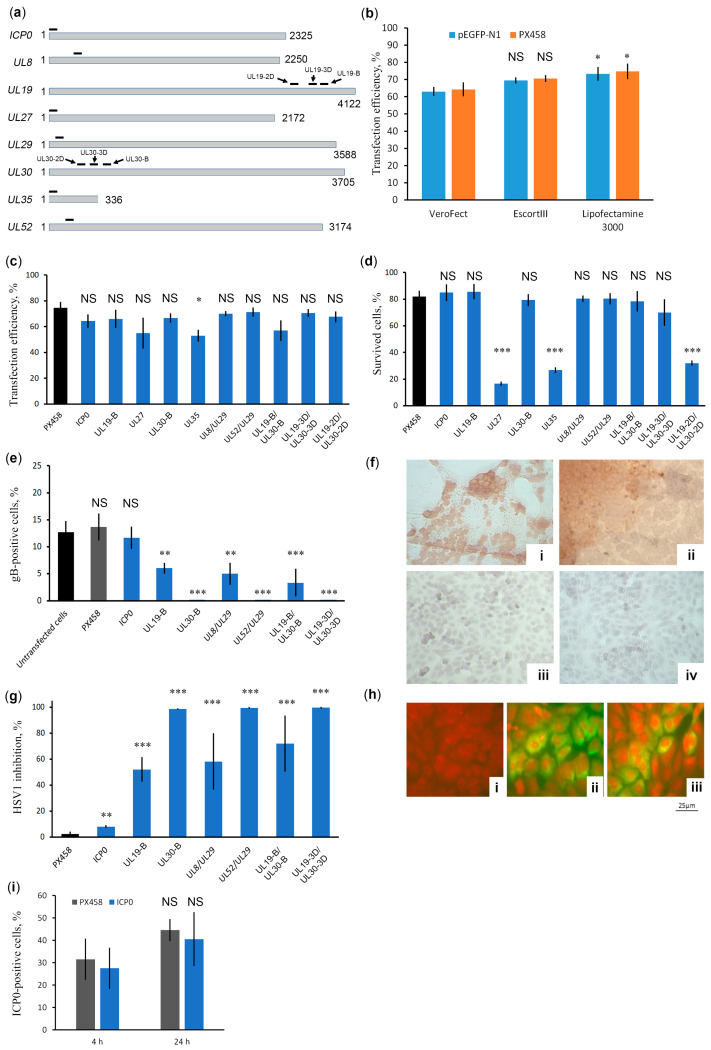
Testing the anti-HSV1 activity of CRISPR/Cas9 plasmids targeting important viral genes. (**a**) Position of spacers for the CRISPR/Cas9 system used in this work. (**b**) Transfection efficiency using different transfection reagents. Data are mean ± SD (*n* = 3). Statistical significance: NS is nonsignificant; * 0.01 < *p* < 0.05, according to Student’s *t* test. (**c**) Transfection efficiency of CRISPR/Cas9 plasmids. Data are mean ± SD (*n* = 3). Statistical significance: NS is nonsignificant; * 0.01 < *p* < 0.05, according to Student’s *t* test. (**d**) Cell toxicity of CRISPR/Cas9 plasmids was measured using the MTT test. Data are mean ± SD (*n* = 3). Statistical significance: NS is nonsignificant *** *p* < 0.001, according to Student’s *t* test. (**e**) Anti-HSV-1 activity of CRISPR/Cas9 plasmids was assessed by gB staining with mAbs at 2 dpi. Data are given as mean ± SD (*n* = 3). Statistical significance: NS is nonsignificant ** 0.001 < *p* < 0.01; *** *p* < 0.001, according to Student’s *t* test. (**f**) Characteristic photographs of cell cultures infected with HSV-1 at 2 dpi: (**i**) untransfected culture, (**ii**) cells transfected with empty vector PX458, (**iii**) cells transfected with CRISPR/Cas9 plasmid with UL19/UL30-B double spacers, (**iv**) cells transfected with CRISPR/Cas9 plasmid with UL19/UL30-3D double spacers. Magnification 100×. (**g**) Evaluation of HSV-1 inhibition rate in Vero cell cultures transfected with CRISPR/Cas9 plasmids. Data are mean ± SD (*n* = 3). Statistical significance: ** 0.001 < *p* < 0.01; *** *p* < 0.001, according to Student’s *t* test. (**h**) ICP0 staining of Vero cells: (**i**) native, uninfected Vero cells; (**ii**) untransfected Vero cells infected with HSV-1; (**iii**) infected Vero cells transfected with CRISPR/Cas9 plasmid targeting *ICP0*. ICP0 protein was determined by immunofluorescence using anti-ICP0 antibodies conjugated with FITC (green staining) followed by staining of cells with Evans blue dye (red staining). Magnification 400×. (**i**) Estimation of the proportion of ICP0-producing cells at the initial stages of HSV-1 infection in the presence of CRISPR/Cas9 plasmid targeted *ICP0*. Data are mean ± SD (*n* = 3). Statistical significance: NS is nonsignificant, according to Student’s *t* test.

In our study, we targeted the plasmid-expressed CRISPR/Cas9 system against those HSV-1 genes that have previously been found to be relatively effective targets for CRISPR/Cas systems delivered in lentiviral constructs, meganucleases delivered in AAV vectors or genes that are supposedly important for HSV-1 infection in vivo. These genes are the *ICP0, UL8, UL19, UL27, UL29, UL30, UL35*, and *UL52* genes (Table 1). The spacers targeting these genes (their positions are marked in Figure 1a) were cloned into vector PX458 individually (against *ICP0, UL19, UL27, UL30,* and *UL35*) or in pairs (UL8/UL29, UL52/UL29, UL19-B/UL30-B, UL19-2D/UL30-2D, UL19-3D/UL30-3D).

Before the main experiments, we evaluated the efficiency of transfection with several transfection reagents using GFP-expressing plasmids (Figure 1b). Our data show that Lipofectamine 3000 is the most effective transfection reagent. All three reagents contain polycationic lipids that form nanoparticles that bind to plasmid DNA and entrap it. The reagents differ in additives: EscortIII contains a neutral non-transfection lipid compound, Verofect contains a polymer, and Lipofectamine 3000 is used in combination with the P3000 enhancer reagent. These additives can affect not only the fusion of nanoparticles with the cell membrane, but also the subsequent biological processes, provide DNA protection from lysosomal enzymes, and mediate efficient plasmid transport to the nucleus. Obviously, the higher the transfection efficiency, the more effective the CRISPR/Cas9-mediated protection of cells against HSV-1 infection will be. Therefore, Lipofectamine 3000 was used in subsequent experiments. Next, our data show that all CRISPR/Cas9 plasmids transfect the Vero cell line with a similar efficiency of 55% and 75% (Figure 1c), but differ greatly in cellular toxicity (Figure 1d). Because we used the same vector backbone for spacer cloning, the reason for the cytotoxicity of CRISPR/Cas9 plasmids with spacers UL27, UL35, and UL19-2D/UL30-2D is due to the off-target effects of Cas9. Therefore, we tested the anti-HSV-1 activity of those individual or paired spacers that showed the least cytotoxicity. Two days post infection (2 dpi) of cell cultures transfected with CRISPR/Cas9 plasmids, anti-HSV-1 activity was tested in two experiments. In the first experiment, we counted the proportion of gB-positive cells, that is, cells maintaining active HSV-1 replication (Figure 1e). Panels i and ii of Figure 1f show a typical pattern of intense brown staining of gB-expressing HSV-1-positive cells at 2 dpi Vero cultures that are not transfected with any CRISPR/Cas9 plasmids or transfected with plasmids that do not target or ineffectively target HSV-1 genes. Panels iii and iv of Figure 1f show a typical pattern of no gB-positive cells at 2 dpi Vero cultures transfected with highly efficient CRISPR/Cas9 plasmids. In the second experiment, we estimated the rate of production of infectious HSV-1 particles (Figure 1g). Unexpectedly, *ICP0* targeting had only a minor effect on HSV-1 replication or HSV-1 infection rate. The UL30-B, UL52/UL29, and UL19-3D/UL30-3D (containing spacers UL19-3D and UL30-3D) spacers controlled HSV-1 infection most effectively (Figure 1e,g). The spacers in the UL19-B/UL30-B pair (containing spacers UL19-B and UL30-B) target the same *UL19* and *UL30* genes as the UL19-3D/UL30-3D pair, but have different locations. These results clearly show that the targeting efficiency of viral genes by CRISPR/Cas systems strongly depends on their location. Our results also show that a single spacer against *UL30* can effectively control HSV-1 infection for at least 2 dpi.

Since the CRISPR/Cas plasmid targeting the *ICP0* gene has little effect on the infectivity of HSV-1, we tested the presence of the ICP0 protein. Specific ICP0 staining revealed no significant differences in the number of cells producing ICP0 at either the late (24 h) or earlier (4 h) stages of HSV1 infection (Figure 1(hi)). These data suggest that the virus surviving the action of the CRISPR/Cas9 system produces the ICP0 protein. It is known that viruses can escape the action of CRISPR/Cas9 using NHEJ mutagenic repair [39,40]. Because ICP0 is critical to the HSV-1 lytic infection process [41] surviving clones can bear indels in the CRISPR/Cas9 target site that do not disrupt the ICP0 mRNA reading frame. Therefore, we harvested the virus from cells transfected with the CRISPR/Cas9 system targeting *ICP0* and checked the sequence of the *ICP0* target region by PCR followed by sequencing. It turned out that the sequence of the *ICP0* target region did not change. This contradicts previous reports of effective targeting of the *ICP0* gene [29]. HSV-1 has two *ICP0* genes, and these results can be explained by highly active homologous recombination, which immediately repairs Cas9-induced damage in both copies of the *ICP0* gene. We cannot rule out the possibility that such efficient *ICP0* repair may be a specific feature of our strain. However, a simple explanation is that the spacer used is ineffective, possibly due to the dense arrangement of nucleosomes in the target region. The inhibitory effect of chromatin structure on CRISPR/Cas9 system activity has been well described [42,43].

### 2.3. CRISPR/Cas9 System against UL30 Provides Long-Term Protection against HSV-1 Infection

**Figure 2:**
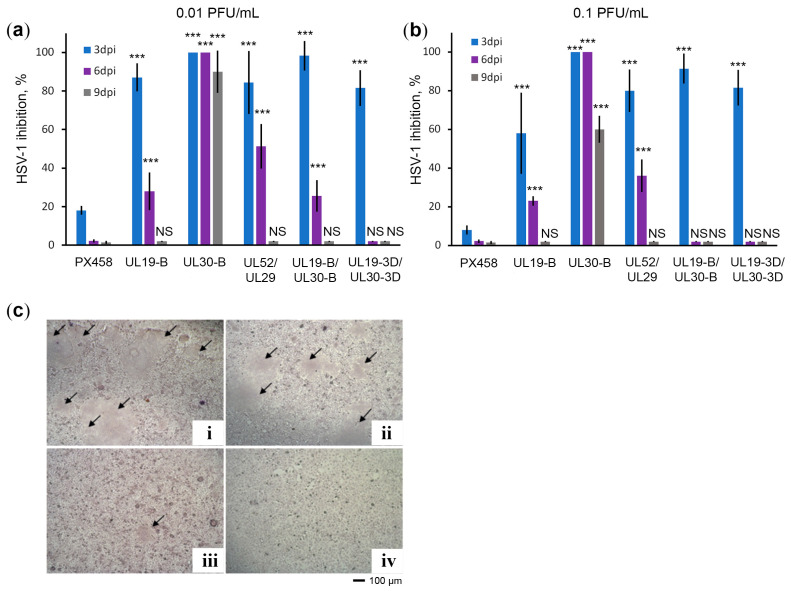
The CRISPR/Cas9 system with a single *UL30* spacer completely inhibited HSV-1 infection for at least 6 days. (**a**) Rate of HSV-1 inhibition by CRISPR/Cas9 plasmids in Vero cultures infected with HSV-1 at a concentration of 0.01 PFU/mL. Data are mean ± SD (*n* = 3). Statistical significance: NS–nonsignificant; *** *p* < 0.001, according to Student’s *t* test. (**b**) Rate of inhibition of HSV-1 by CRISPR/Cas9 plasmids in Vero cultures infected with HSV-1 at a concentration of 0.1 PFU/mL. Data are mean ± SD (*n* = 3). Statistical significance: NS–nonsignificant; *** *p* < 0.001, according to Student’s *t* test. (**c**) Typical images of Vero cell cultures at 2 dpi infected with supernatants from HSV-1 infected cells at day 6 that were not previously transfected (**i**), transfected with empty vector (**ii**), transfected with plasmid CRISPR/Cas9 targeting *UL30* (**iii**), or uninfected cells (**iv**). Arrows indicate areas of infected cells (plaques).

CRISPR/Cas9 plasmids that effectively control HSV-1 infection at 2 dpi were evaluated for the duration of the antiviral effect. For this purpose, Vero cells were infected with HSV-1 at concentrations of 0.01 PFU/mL and 0.1 PFU/mL and the production of infectious HSV-1 particles was assessed on days 3, 6, and 9 of cell culture. The results (Figure 2) show that all CRISPR/Cas9 plasmids were relatively effective in inhibiting HSV-1 infection on day 3 (Figure 2a,b). We observed the most significant differences on days 6 and 9. The CRISPR/Cas9 plasmid targeting *UL30* with the UL30-B spacer appeared to be the most effective. This construct completely inhibits HSV-1 replication in Vero culture for at least 6 days, and on day 9 it inhibits HSV-1 infection by 60–95%, depending on the initial HSV-1 concentration.

In contrast, the CRISPR/Cas9 plasmid UL19-B/UL30-B encoding dual sgRNAs, which contains the same UL30-B spacer, cannot suppress HSV-1 infection at 6 and 9 days. However, we noticed that the UL19-B spacer of this pair is not so effective (Figure 2a,b). Thus, we hypothesized that the ineffective UL19-B spacer titrates the Cas9 protein, thereby reducing the number of highly efficient Cas9/UL30-B sgRNA complexes, and hence the level of cell protection against HSV-1 is reduced.

### 2.4. CRISPR/CasX System against UL30 Efficiently Protects Cells from HSV-1 Infection

**Figure 3:**
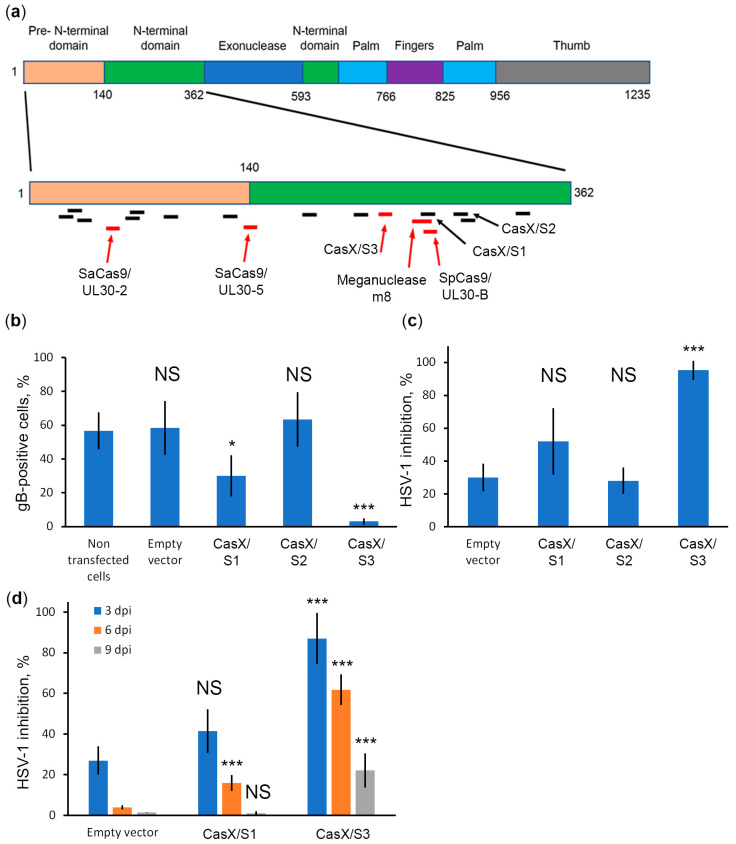
*UL30*-targeted CRISPR/CasX system effectively controls HSV-1 infection in the Vero cell line. (**a**) Matching the positions of the targets of different genomic editors with the UL30 domains. Information about the structure of UL30 was taken from [46]. Targets highlighted in red are considered efficient, targets highlighted in black are considered inefficient. Corresponding genomic editors and spacer names are indicated for effective targets as well as for the CRISPR/CasX system. (**b**) Anti-HSV-1 activity of CRISPR/CasX plasmids was assessed by gB staining at 2 dpi. Data are given as mean ± SD (*n* = 3). (**c**) Evaluation of HSV-1 inhibition rate in Vero cell cultures transfected with CRISPR/CasX plasmids. Data are given as mean ± SD (*n* = 3). (**d**) Long-term inhibition of HSV-1 by CRISPR/CasX plasmids in Vero cultures. Cells were infected with HSV-1 at a concentration of 0.1 PFU/mL. Data are mean ± SD (*n* = 3). Statistical significance: NS–nonsignificant; * 0.01 < *p* < 0.05; *** *p* < 0.001, according to Student’s *t* test.

The Cas9 editor is well characterized and highly active in genome editing. However, it is a large protein, which makes it difficult to efficiently deliver in viral vectors such as adenovirus-associated vectors. In addition, its relatively high rate of off-targeting among other editors raises safety concerns. Therefore, we decided to test the anti-HSV-1 activity of CasX (Cas12e) from *Deltaproteobacteria* (DpbCasX) [44], which is one of the smallest and most accurate genome editors to date [45]. We designed three spacers S1, S2, and S3 targeting *UL30* in the region where the most effective UL30-B spacer target is located (Figure 3a). All plasmids showed no significant cytotoxicity. Spacer S3 showed the greatest anti-HSV-1 activity (Figure 3b,c) and was relatively effective in controlling HSV-1 infection for 3 days (at 90%, Figure 3d). To our knowledge, we were the first to report effective inhibition of HSV-1 infection with CasX nuclease. These data suggest that CasX is a promising genome editor that could be used to develop novel therapeutic approaches for controlling HSV-1 and possibly other herpesvirus infections.

We also compared the positions of known targets in *UL30* (Figure 3a). To our knowledge, all genomic editors have targeted regions of this gene encoding the pre- and N-terminal domains of DNA polymerase [26,36,37]. The target of the highly efficient UL30-B spacer overlaps with the meganuclease m8 target, indicating that this site is highly susceptible to targeting. However, the inefficient CasX S1 spacer also overlaps with this site, suggesting that the CRISPR/CasX system (in addition to different PAMs) has different requirements for the context of the genome for effective targeting. Taken together, these data show that although *UL30* is an important HSV-1 gene, it has a set of CRISPR/Cas-resistant regions and a set of CRISPR/Cas-sensitive regions. Certainly, the regions of the gene that are highly sensitive to CRISPR/Cas activity are critical for DNA polymerase function, and thus for HSV-1 replication.

### 2.5. Cas9 without sgRNA Attenuates HSV-1 Infections

**Figure 4:**
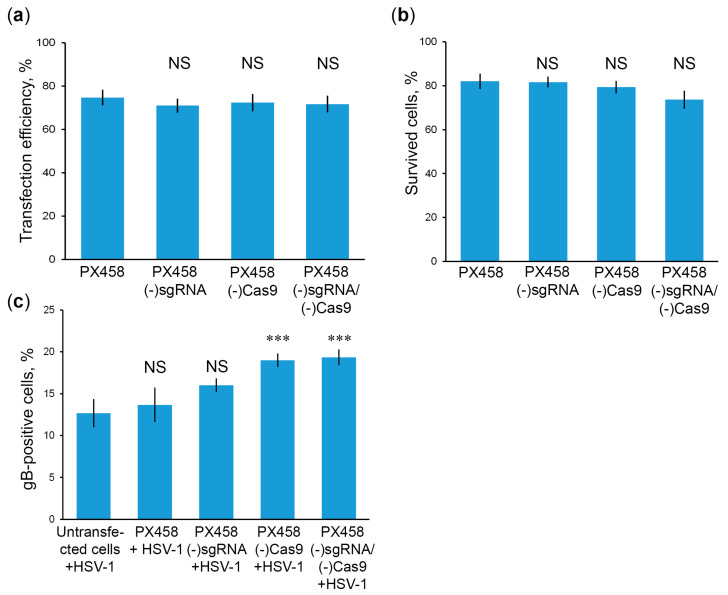
CRISPR/Cas9 components contribute to attenuation of HSV-1 infection. (**a**) Efficiency of transfection of plasmids with disrupted CRISPR components; (**b**) Proportion of surviving cells after transfection with mutant CRISPR plasmids; (**c**) Proportion of gB-positive cells in cultures transfected with CRISPR plasmids at 2 dpi. Data are mean ± SD (*n* = 3). Statistical significance: NS–nonsignificant; *** *p* < 0.001, according to Student’s *t* test.

In our previous work [32], we observed that the CRISPR/Cas9 plasmid without spacers against HSV-1 has some protective effect for cells against HSV-1 infection. It has previously been shown that components of the CRISPR/Cas9 system can trigger an intracellular antiviral response [47]. Therefore, we hypothesize that sgRNA and/or Cas9 themselves have some protective properties against HSV-1. To test this assumption, we infect HSV-1 in Vero cell lines transfected with plasmids lacking the sgRNA gene (PX458(-)sgRNA), or CMV promoter to disrupt Cas9 expression (PX458(-)Cas9), or both the sgRNA gene and CMV promoter (PX458(-)sgRNA/(-)Cas9). According to the results obtained, mutation of CRISPR/Cas9 components had no significant effect on transfection efficiency (Figure 4a) or cytotoxicity of mutant plasmids (Figure 4b). At the same time, the number of gB-positive cells at 2 dpi was significantly increased in the presence of plasmids PX458(-)Cas9 or PX458(-)sgRNA/(-)Cas9 (Figure 4c). Deletion of the only sgRNA backbone also increases the number of gB-positive cells, but the differences are not statistically significant. These results suggest that Cas9 without sgRNA makes a major contribution to the attenuation of HSV-1 infection.

## 3. Discussion

We found highly efficient targets for the CRISPR/Cas9 and CRISPR/CasX systems in the *UL30* HSV-1 gene. A plasmid expressing CRISPR/Cas9 with the UL30-B spacer completely suppresses HSV-1 replication for at least 6 days. To date, this is the longest duration of CRISPR-mediated cell protection against herpesvirus infection.

Earlier studies using CRISPR/Cas9 systems expressed from lentiviral constructs show that at least two spacers are required to effectively control HSV-1 infection, providing deletion of a fragment of the HSV-1 genome [26,29,30]. Single spacers lead to viral escape and CRISPR/Cas resistance due to NHEJ-mediated target modification [48].

Recently, it has been shown that there may be individual Cas12a spacers capable of completely eradicating HIV [40,49]. The superior efficacy of the antiviral activity of Cas12a may be due to its mechanism of action. Cas12a cuts the target in the region distal to the PAM that is well tolerated by mismatches, so the enzyme can attack the mutated target again after NHEJ repair and thus act in repetitive cycles. This mode of action of Cas12a is supported by the observation that Cas12a usually produces longer deletions than Cas9 [40].

However, we found a site in the essential HSVI-1 gene *UL30* encoding a viral DNA polymerase, which targeting with Cas9 and the UL30-B spacer provides the longest-lasting protection for Vero cell culture. Earlier studies show that the efficacy of suppressing HSV-1 infection with *UL30* targeting varies greatly. Some studies show its ineffectiveness [26], while others suggest that it is a good target for inhibition of HSV-1 infection [36,37]. We hypothesize that there may be sites within the *UL30* gene that can be mutated by NHEJ after Cas9 cleavage, leading to a more or less functional enzyme, but there may also be sites critical to enzyme function, so that any NHEJ mutation would lead to substantial or complete inactivation of the enzyme. Thus, the CRISPR/Cas9 system can be used to map critical sites, such as UL30-B, to select good targets for future CRISPR/Cas-mediated therapies for HSV-1 infection. Moreover, given the relatively high off-target activity of CRISPR/Cas systems, it is safer to use one spacer instead of two or more in therapeutic approaches.

The highly variable efficacy of CRISPR/Cas spacers leads us to speculate that the context of the genome may critically influence the efficiency of HSV-1 targeting. In the case of SaCas9 from *Staphylococcus aureus*, it has been shown that even in vitro some sgRNAs are ineffective [37], suggesting that viral DNA sequences may be resistant to Cas9 cleavage. The HSV-1 genome is known to be enriched in G-quadruplex (G4) clusters [50]. Recently, G4s have been shown to significantly reduce the cleavage activity of Cas9 [51]. At present, G4s have been described in HSV-1 regulatory regions, and it is unknown whether the coding regions can also form G-quadruplexes or other alternative DNA structures that can interfere with the action of CRISPR/Cas systems. Nevertheless, the property of GC-rich DNA to form Cas-resistant structures makes it difficult to find highly efficient spacers for herpesviruses.

In our previous and present studies, we observed complete suppression of HSV-1 infection in Vero cell cultures, despite the fact that about 75% of cells received the CRISPR/Cas plasmid and about 25% of cells did not. Earlier results show that sgRNA can induce a type I interferon response in cultured human cells [47]. In turn, interferons control HSV-1 infection [52]. These data suggest that the CRISPR/Cas system can nonspecifically activate immune responses in transfected cells, which in turn produce output signals that cause neighboring untransfected cells to be ready to resist HSV-1 invasion. However, our results show that sgRNA scaffold plays only a minor role in protecting cells from HSV-1 invasion. Cas9 is known to be toxic to prokaryotes with GC-rich genomes even in its dead nuclease form (dCas9) [53,54]. Since Cas9 has a GC-rich PAM, its toxicity may be due to nonspecific binding to PAM-like sequences. Indeed, a nontoxic version of dCas9 was created by eliminating its PAM-binding activity [55]. Therefore, we hypothesize that Cas9 can nonspecifically attenuate HSV-1 replication by binding to PAMs throughout the HSV-1 genome. However, the nonspecific action of the CRISPR/Cas system can only slightly attenuate HSV-1 infection, indicating a more effective way(s) to protect untransfected cells from HSV-1. It is well known that various mammalian cells are capable of forming extracellular vesicles [56]. Earlier results show that Cas9/sgRNA complexes efficiently load into these vesicles even without any additional modifications, indicating some intrinsic affinity of the Cas9/sgRNA complex for extracellular vesicles [57]. Using these data, we hypothesized that cells successfully transfected with the CRISPR plasmid produce extracellular vesicles loaded with Cas9/sgRNA complexes, and these vesicles deliver Cas9/sgRNA complexes to untransfected cells, making them resistant to HSV-1.

The limitations of the study are as follows. The Cas9 genomic editor provides excellent efficacy in suppressing HSV-1 infection, but it has high off-target activity and large size. The CasX genomic editor is the most specific and smallest of the known genomic editors, but it is less active than Cas9. Both systems are effective in in vitro systems with the Vero cell line culture, and further work is needed to optimize the delivery of CRISPR/Cas systems and test their activity in an in vivo model.

In conclusion, CRISPR/Cas9 and CRISPR/CasX systems targeting critical regions of the *UL30* gene can provide effective long-term suppression of HSV-1 infection in vitro. The CRISPR/CasX system promises to be a relatively effective and safe therapeutic approach for herpesvirus infections.

## 4. Materials and Methods

### 4.1. Cells and Viral Strain

Green monkey kidney cells (Vero) were obtained from the collection of cell cultures of the Gamaleya National Research Center for Epidemiology and Microbiology, Ministry of Health of the Russian Federation. Cells were cultivated in the Eagle MEM medium with the addition of 10% fetal bovine serum (FBS), 2 mM L-glutamine, 50 μg/mL gentamicin (all reagents were purchased from PanEco LLC, Moscow, Russia). Cells were cultured at 37 °C in an atmosphere of 5% CO2. Molecular cloning was performed using chemocompetent cells of *E. coli* strain XL1Blue (Eurogen, Moscow, Russia). HSV-1 strain “US” was obtained from the State virus collection of the Gamaleya National Research Center for Epidemiology and Microbiology of the Ministry of Health of the Russian Federation. The virus was multiplied in Vero cells using standard culturing methods. Viral titers were determined by plaque assay on confluent Vero cell cultures. Foci of infected cells (plaques) were detected and counted using an inverted microscope Primovert (Zeiss, Oberkochen, Germany). The virus titer was determined according to the formula: *A* = *ab/v*, where *A* is the number of plaque-forming units per cell (PFU/cell); *a* is the average number of plaques per well; *b* is the dilution of the virus; *v* is the volume of virus-containing material added. The infectious titer of the virus used in the experiments was 6 × 10^7^ PFU/mL.

### 4.2. Purification of HSV-1

HSV-1 was purified according to the protocol described in [58] with some modifications. Briefly, Vero cells were incubated in 175 cm^2^ culture flasks. After reaching a monolayer, the cells were infected with HSV-1 at a dilution of 1/1000. After maximum virus-specific cytopathic effect was manifested, 1 freeze–thaw cycle was performed. The virus-containing fluid was centrifuged at 13,000× *g* (rotor SW32.1, Beckman Coulter, Brea, CA, USA) for 20 min to remove residual cells. The supernatant was then ultracentrifuged at 123,000× *g* for 1 h to precipitate the virus. The precipitate was resuspended in 1 mL of 10 mM Tris-HCl, pH 7.4, and left overnight at room temperature. In a centrifuge tube, 2 mL of 60% sucrose, followed by 10 mL of 15% sucrose and the virus suspension were placed. Ultracentrifugation was then performed for 1 h at 123,000× *g*. Then, virus particles concentrated in the 60% sucrose layer were collected and inactivated by heating at 56 °C for 30 min.

### 4.3. DNA Purification from HSV-1 Preparations

HSV-1 genomic DNA was isolated according to the protocol described in [59], with minor modifications. Briefly, 300 μL of HSV-1 particle concentrate was mixed with 300 μL of extraction buffer (10 mM Tris-HCl (pH 8), 10 mM EDTA, 100 mM NaCl, 1% SDS) and 20 μL of proteinase K (20 mg/mL). The mixture was incubated overnight at 37 °C. Then, 620 μL of a buffered (pH 8.0) phenol/chloroform/isoamyl alcohol solution (25:24:1) was added and mixed by pipetting. The phases were separated by centrifugation at 21,000× *g* for 10 min at room temperature. The aqueous phase was transferred to a new tube and mixed with 3 volumes of 95% ethanol and 1/10 volume of 3 M sodium acetate (pH 5.2) and incubated at −20°C overnight. Nucleic acids were precipitated by centrifugation at 21,000× *g* for 10 min at room temperature. The precipitate was treated with RNase A to remove RNA. The DNA was precipitated again with ethanol and sodium acetate.

### 4.4. Sequencing of the HSV-1 Genome

Genomic DNA (0.1 µg) purified from HSV-1 preparations was used to prepare sequencing library with the Nextera DNA Flex Library Preparation Kit (Illumina, San Diego, CA, USA) according to the manufacturer’s protocol. Size of the genomic library was analyzed on an Agilent 2100 Bioanalyzer (Thermo Fisher Scientific, Waltham, MA, USA) was about 600 bp. The library was sequenced on an Illumina MiSeq System using the MiSeq Reagent Micro Kit v2 (300 cycles). Primary analysis of the raw reads was performed using the Illumina software (https://basespace.illumina.com/dashboard, accessed on 31 August 2021). A draft HSV-1 genome was de novo assembled using the Velvet software with default parameters (https://basespace.illumina.com/apps/8556549/Velvet-de-novo-Assembly?preferredversion, accessed on 31 August 2021).

### 4.5. Cloning of Spacers into Plasmids of the CRISPR/Cas System

Spacers against target HSV-1 genes were designed using CRISPOR software (http://crispor.tefor.net/, accessed on 27 September 2021) with default parameters. To produce plasmids of CRISPR/Cas9 system, spacers in the form of oligonucleotides or PCR products obtained oligonucleotide pairs UL19-Best-F/UL30-Best-F, UL19-2nd-F/UL30-2nd-F, and UL19-3d-F/UL30-3d-F (Table 2) using AIO-mCherry (Addgene (www.addgene.org, accessed on 27 September 2021), cat.no 74120) as a template were cloned into pSpCas9(BB)-2A-GFP (PX458) or pSpCas9(BB)-2A-Puro (PX459) vectors (Addgene, cat. no. 48138 and 48139, respectively) cut at BbsI sites. To produce plasmids of CRISPR/CasX system, fragments of pBLO 62.4 plasmid was amplified with pairs of oligonucleotides U6-PciI-F/CasX-S1-sgRNA-R, U6-PciI-F/CasX-S2-sgRNA-R, and U6-PciI-F/CasX-S3-sgRNA-R and cloned into pBLO 62.4 vector cut at PciI and KpnI sites. The correctness of the cloned guide RNA spacers was confirmed by sequencing with the U6-ch-F oligonucleotide. All plasmids were purified from *E. coli* using the PureLink™ HiPure Plasmid Midiprep Kit (Thermo Fisher Scientific, Waltham, MA, USA) to obtain endotoxin-free preparations for the Vero cell line transfection.

### 4.6. Vero Cells Line Transfection

Vero cells were transfected using Lipofectamine 3000 Reagent (Thermo Fisher Scientific, Waltham, MA, USA) or VeroFect (OZ Biosciences, San Diego, CA, USA), or EscortIII (Sigma, St. Louis, MO, USA) according to the manufacturers’ recommendations. Cells were incubated in a 96-well plate at a concentration of 2 × 10^5^ cell/mL in DMEM growth medium without antibiotics. After 24 h, a mixture of test plasmids and transfecting agents was added to the cells in a 1:2 ratio. The efficiency of transfection was assessed by GFP fluorescence after 48 h using an AxioScopeA1 inverted fluorescent microscope (Zeiss, Oberkochen, Germany). The ratio of the number of fluorescent cells expressing GFP to the total number of cells in the population was calculated and expressed as a percentage.

### 4.7. Evaluation of the Cytotoxicity of CRISPR/Cas Constructs

The cytotoxicity of CRISPR/Cas plasmids was assessed by MTT-test. Vero cells were transfected, after 24 h the cells were washed and the growth medium was added. After 72 h, cells were treated with 50 μL MTT solution (5 mg/mL 3-(4,5-dimethylthiazol-2-yl)-2,5-diphenyltetrazolium in Eagle’s MEM) for 3 h at 37 °C. After 3 h, the medium was removed and replaced with 100 μL of MTT solvent (0.1N HCl in isopropanol), and the cells were placed on a shaker for 10 min. MTT activity was measured using a Multiskan microtiter plate reader (TECAN, Mannedorf, Switzerland) at 570 nm and a reference wavelength of 690 nm.

### 4.8. Detection of HSV-1

HSV-1 infected cells were detected by immunocytochemical staining. Cells were fixed with chilled methanol, subsequently incubated with the murine monoclonal antibodies (mAbs) against the late structural protein gB of HSV-1 (ab6506; Abcam, Cambridge, United Kingdom) for 1 h and the horseradish peroxidase-conjugated anti-mouse secondary antibody (PO260; Dako, Glostrup, Denmark) for 1 h. Then, a solution of 3,3′-diaminobenzidine at a concentration of 1 mg/mL in 0.05 M Tris-HCl buffer (pH 7.4) with 3% hydrogen peroxide was added. The reaction was stopped after 10 min by adding distilled water to the wells. The results were evaluated using an AxioVertA1 inverted microscope. The number of stained cells containing gB HSV-1 protein was counted and presented as a percentage of the total number of cells in the population.

To assess the number of newly formed viral particles in cells transfected with CRISPR/Cas plasmids, culture fluid was taken and added in to uninfected Vero cells monolayer. The presence of gB-positive cells was determined immunocytochemically as described above.

### 4.9. Estimation of HSV-1 Inhibition Rate

Culture fluids from infected cells pre-transfected with CRISPR/Cas9 plasmids were taken and added to Vero cells monolayer. After 1, 3, 6, or 9 days the number of plaques was analyzed using an AxioVert A1 inverted microscope (Zeiss Oberkochen, Germany). The HSV-1 inhibition rate was calculated using the following formula: HSV-1 inhibition (%) = 100 − [(TC/CC) × 100], where TC and CC denote the number of plaques in culture medium from transfected cells and control cells infected with HSV-1, respectively.

### 4.10. ICP0 Protein Detection

Vero cells transfected with CRISPR/Cas9 plasmid targeted *ICP0* gene, infected with HSV-1 at 0.1 PFU/cell after 3 days. At 4 h and 24 h after infection, cells were fixed with methanol for 20 min at −20°C, washed with PBS, layered with mAbs to the ICP0 protein of HSV-1 (Anti-HSV-1 ICP0 antibody Abcam, ab6513, Cambridge, UK), and incubated for 1 h at 37 °C. After wash with PBS, FITC-labeled anti-mouse antibody (Rabbit Anti-Mouse IgG H&L (FITC) Abcam, ab6724, Cambridge, UK) was added, and incubated for 30 min at 37 °C. Infected and uninfected Vero cells were used as controls. The results were recorded using an AxioScop fluorescence microscope (Zeiss, Oberkochen, Germany).

### 4.11. Statistical Analysis

Statistical processing of the results was performed using GraphPadPrism 5.01. Statistical significance of differences between the mean values was determined using a two-way unpaired *t*-test (Student’s *t*-test). Differences were considered statistically significant at *p* < 0.05.

## Figures and Tables

**Table 1:** Characteristics of HSV-1 genes used as targets for CRISPR/Cas9 system.

Gene Name	Description	References
*ICP0*	RING-type E3 ubiquitin ligase, acts on initial stages of infection, or during HSV-1 reactivation, helps to evade cellular antiviral response	[29]
*UL8*	DNA helicase/primase complex-associated protein	[26,30]
*UL19*	Major capsid protein, forms an icosahedral capsid with a T = 16 symmetry consisting of 162 capsomers.	[36]
*UL27*	gB, envelope glycoprotein that forms spikes at the surface of virion envelope, essential for the initial attachment to the host cell receptors, involved in fusion of viral and cellular membranes, together with gK induces syncytia formation	[26,33]
*UL29*	ssDNA-binding protein	[26,30,37]
*UL30*	Large catalytic subunit of DNA-directed DNA polymerase, essential for HSV-1 genome replication	[26,36,37]
*UL35*	Small capsomere-interacting protein, participates in the assembly of the infectious particles, forms a layer between the capsid and the tegument	[38]
*UL52*	DNA primase, essential for HSV-1 genome replication	[26]

**Table 2:** Oligonucleotides used in the work.

Name of the Oligonucleotide	Sequence (5′→3′)	Purpose
ISP0_F	CACCTCCCTGCGACCGAGACCTGC	Cloning spacers for CRISPR/Cas9 system
ISP0_R	AAACGCAGGTCTCGGTCGCAGGGA	
ICP0_ch_F	AACTCGTGGGCGCTGATTGA	
ICP0_ch_R	TCGTCGCTCCCCCCGTCCTCT	
UL27_F	CACCGGTGCCGGTGGTTCGTCGTA	
UL27_R	AAACTACGACGAACCACCGGCACC	
UL35_F	CACCGTGAAATTGCGGGACGGCCAT	
UL35_R	AAACATGGCCGTCCCGCAATTTCAC	
UL35_ch_F	AAGGACGCACCGCCGCCCTA	
UL35_ch_R	CGGCCCCTTGGGTGCCCTGG	
UL27_ch_F	GGAGCCGCCGACGCCACCAGG	
UL27_ch_R	CGTACGACTCCGACTGTCCGCT	
U6-ch-F	CGATACAAGGCTGTTAGAGAGA	
UL19-B-F	ATATAGAAGACCTCACCGTAGTTGACGTCGGTCGACACGTTTTAGAGCTAGAAATAGCAAG	
UL19-2D-F	ATATAGAAGACCTCACCGGACCGCGTTCCGCAGGTACAGTTTTAGAGCTAGAAATAGCAAG	
UL19-3D-F	ATATAGAAGACCTCACCGTAAACTCACACACGGCATCCGTTTTAGAGCTAGAAATAGCAAG	
UL30-B-R	TAGAGGAAGACCCAAACCTTCGGACGTAGACGCGGTACGGTGTTTCGTCCTTTCCAC	
UL30-2D-R	TAGAGGAAGACCCAAACCGTGCCGTAAACGTGAACGGCGGTGTTTCGTCCTTTCCAC	
UL30-3D-R	TAGAGGAAGACCCAAACGGCGCGTCGTTCCGCGGCATCGGTGTTTCGTCCTTTCCAC	
UL30-BsgRNA-F	CACCGTACCGCGTCTACGTCCGAAG	
UL30-BsgRNA-R	AAACCTTCGGACGTAGACGCGGTAC	
UL19-B-sgRNA-F	CACCGTAGTTGACGTCGGTCGACAC	
UL19-B-sgRNA-R	AAACGTGTCGACCGACGTCAACTAC	
U6-PciI-F	TTTTGCTCACATGTGAGGGCCTATTTCC	Cloning spacers for CRISPR/CasX system
CasX-S1-sgRNA-R	TATGTAACGGGTACCAAAAAAAATGTTTTACCGCGTCTACGTCCTTTGATGCGTTTTACTTATCGGTTTC	
CasX-S2-sgRNA-R	TATGTAACGGGTACCAAAAAAAACAACTTCTGCCCGGCCATCACTTTGATGCGTTTTACTTATCGGTTTC	
CasX-S3-sgRNA-R	TATGTAACGGGTACCAAAAAAAAGCGCTCCACCACCTCCGCCTCTTTGATGCGTTTTACTTATCGGTTTC	

## Data Availability

The datasets generated during and/or analyzed during the current study are available from the corresponding author on reasonable request.

## Supplementary Materials

The supporting information can be downloaded at: https://www.mdpi.com/article/10.3390/ijms232314847/s1.
